# Bridging the Maize Yield Gap: Empirical Evidence on the Adoption of BH660 Hybrid Variety in West Gojjam, Ethiopia

**DOI:** 10.1155/tswj/1556629

**Published:** 2026-07-28

**Authors:** Belete Animaw, Silabat Enyew, Gebyaw Demeke

**Affiliations:** ^1^ College of Business and Economics, Debre Markos University, Debre Markos, Ethiopia, dmu.edu.et

**Keywords:** adoption, Ethiopia, improved maize BH660, logit, smallholder farmers

## Abstract

The adoption of agricultural technology by smallholder farmers plays a pivotal role in accelerating Ethiopia′s economic transformation, particularly within the agricultural sector. Despite government policy initiatives that actively support agricultural technology, there is still a significant adoption gap among smallholder farmers because of institutional, environmental, and resource limitations. This study is aimed at exploring the factors influencing the adoption of the improved maize variety, BH660, among smallholder farmers in Womberma district, West Gojjam Zone, Amhara Regional State. The research utilized primary cross‐sectional data collected from a total sample of 370 smallholder farmers. A multistage sampling method was employed, with respondents selected using a probability proportional technique. The collected data were analyzed using a binary logit model to identify the most significant determinants of BH660 adoption. With 79.08% of the observations properly classified (80.85% sensitivity and 77.20% specificity), the empirical model showed a strong fit. The results of the regression analysis showed that BH660 adoption was positively and statistically significantly impacted by gender, educational attainment, and access to training, market information, positive behavioral attitudes, and agricultural experience. In particular, calculated marginal effects showed that the likelihood of adopting the hybrid seed was raised by 43.5% and 28.4%, respectively, by having a favorable behavioral attitude and receiving regular market information. Adoption likelihood was also raised by 25.4%, 28.2%, and 25.5% for men, literacy, and training and by 0.8% for every year of farming experience. Based on these results, regional policy actors can take advantage of these important drivers by putting in place functional adult literacy programs, growing Farmer Training Center activities, and adopting gender‐responsive extension frameworks to effectively increase adoption rates and foster sustainable agricultural development.

## 1. Introduction

Ethiopia remains one of the least developed countries in Africa, with a significant portion of its population relying on agricultural activities, including crop production and animal husbandry. The agricultural sector contributes approximately 80% of the GDP, over 60% of employment, and 70% of export earnings, despite relying on outdated production methods and technologies [1]. Modernizing the agricultural sector is crucial for enhancing the welfare and living standards of Ethiopian citizens. This includes the adoption of advanced technologies such as fertilizers, improved seeds, and pesticides by farmers in rural areas. According to [2], it emphasizes that increasing the productivity of rural farmers can significantly improve living standards and welfare, particularly when supported by the introduction of advanced agricultural technologies. This can be achieved through the dissemination of new pesticides and insecticides or by providing access to high‐yield, improved seeds.

Maize (*Zea mays*), a versatile seed and plant cultivated in numerous species, serves multiple purposes, including the preparation of various maize‐based foods, animal feed, raw materials for the processing industry, soil fertility enhancement, and the production of chemicals, biofuels, and ornamental products [3]. It is a highly nutritious crop that supports bone, kidney, and heart health, offers protection against cancer, boosts red blood cell production, reduces fat accumulation, promotes eye and skin health, and strengthens the immune system. As a high‐value and profitable cereal crop, the economic viability of maize production is influenced by the commercial use of its by‐products, with approximately three‐fourths of the maize produced being consumed at the household level by small‐scale producers [4].

Maize is a crucial crop in terms of access to and application of improved agricultural inputs, including fertilizers, advanced seed varieties, pesticides, herbicides, and superior farming techniques, compared with other cereal crops [5]. Numerous studies have been conducted on the adoption of maize‐related technologies in various regions of Ethiopia [6–8].

On the other hand, [8] investigated factors influencing the adoption of improved maize varieties among male‐headed and female‐headed households in the West Hararghe Zone. Their research utilized cross‐sectional data collected from 148 maize‐producing farmers. A logistic regression model was employed to estimate the likelihood of adopting improved maize varieties. The findings revealed that farm size, livestock ownership (measured in tropical livestock units [TLUs]), and access to extension services had a positive and significant impact on adoption. In contrast, the farmer′s age and the distance to the nearest input market negatively and significantly influenced the probability of adopting improved maize varieties [8].

The research by [5] examined the effects of adopting improved maize varieties on farm productivity and farmer welfare in the East Hararghe Zone of Ethiopia. Utilizing a descriptive propensity score matching approach combined with endogenous switching regression, the study assessed welfare impacts, whereas a stochastic frontier model corrected for sample selection was employed to evaluate farm productivity. The findings indicate that the adoption of improved maize varieties significantly enhances both farmer well‐being and agricultural productivity [5].

The Ministry of Agriculture and Rural Development (MoARD) brought improved maize varieties to the study region, such as BH660, BH540, PHB3250, and BH140 [9]. The Ethiopian Agricultural Research Organization (EARO) and the Ethiopian National Seed Industry tested these cultivars. A thorough evaluation of the variables affecting these types′ uptake in the area is necessary before deciding to adopt them. However, farmers have not entirely or successfully embraced the agronomists′ suggested types of maize. Consequently, the rate and level of adoption continue to be low, resulting in inadequate agricultural production [10].

Despite the significant potential of maize production in Ethiopia, it remains underutilized and has not effectively contributed to alleviating food insecurity and poverty. The average maize productivity in Ethiopia was estimated at 3736 kg/ha, which is considerably lower than the global average of 5818 kg/ha in 2017 [11]. Similarly, in the Amhara National Regional State, the average maize production was reported at 3508 kg/ha during the 2015/2016 period [11].

This study isolates the adoption of better maize varieties, but smallholders often select from integrated technology packages, such as the simultaneous adoption of enhanced seeds, chemical fertilizers, and row‐planting techniques. Due to supply chain limitations, public extension systems frequently release enhanced seeds without supplementary inputs, which is imposed by local institutional frameworks. Before examining input complementarities, it is easier to identify baseline impediments to genetic innovation by isolating the seed component. Simultaneously, this narrow focus creates a structural limitation: While individual inputs frequently show strong economic complementarities, assessing a single component would not adequately convey the multifaceted nature of agricultural modernization. Multivariate probit or double‐hurdle extensions should be used in future studies to capture these sequential and combined adoption processes throughout northwest Ethiopia.

The majority of adoption studies, however, treat smallholders as solely agricultural agents and neglect to examine how nonfarm income diversification interacts with a household′s financial capacity to adopt inputs [8, 10]. Additionally, there is a lack of critical synthesis regarding how institutional barriers such as localized credit access constraints and distinct regional disparities across agroecological zones prevent aggregate agricultural policies from succeeding at the district level [8, 10]. This study addresses these explicit gaps by examining the socioeconomic, institutional, and geographic bottlenecks inhibiting technology uptake specifically in the Womberma district. To address these empirical omissions, the study evaluates two core research questions: The study assesses two main research issues in order to answer these empirical omissions: What are the main institutional and socioeconomic factors influencing smallholder families′ choices to adopt enhanced maize varieties? And when the first barrier choice is reached, how much do adopting households accelerate their adoption? Based on these questions, the study tests two operational hypotheses: The probability of adopting improved maize varieties is significantly and positively increased by access to institutional support, such as agricultural extension services, formal training, credit, market information, and fertilizer; additionally, adoption decisions are expected to be positively influenced by farmers′ educational level, positive attitude toward technology, farm experience, family size, marital status, livestock ownership, farm land size, and annual income. On the other hand, it is hypothesized that socioeconomic and demographic factors, such as the household head′s advanced age, female gender, greater distance to input markets, and larger family size (as a potential resource constraint), will considerably lower the likelihood and intensity of technology adoption.

The paper is structured as follows: Section 2 outlines the data and estimation methods, Section 3 presents and discusses the results, and Section 4 provides the conclusions and recommendations of the study.

## 2. Materials and Methods

West Gojjam, a zone in the Amhara Region of Ethiopia, is situated about 350 km northwest of Addis Ababa and south of Bahir Dar. It is bounded by the Abay River to the south, which demarcates its border with the Oromia and Benishangul‐Gumuz regions. It is bordered by Alefa to the northwest, North Gojjam to the north, East Gojjam to the east, and the Awi Zone to the west. The study area comprises seven rural district administrations and six city administrations [12].

**Figure 1:**
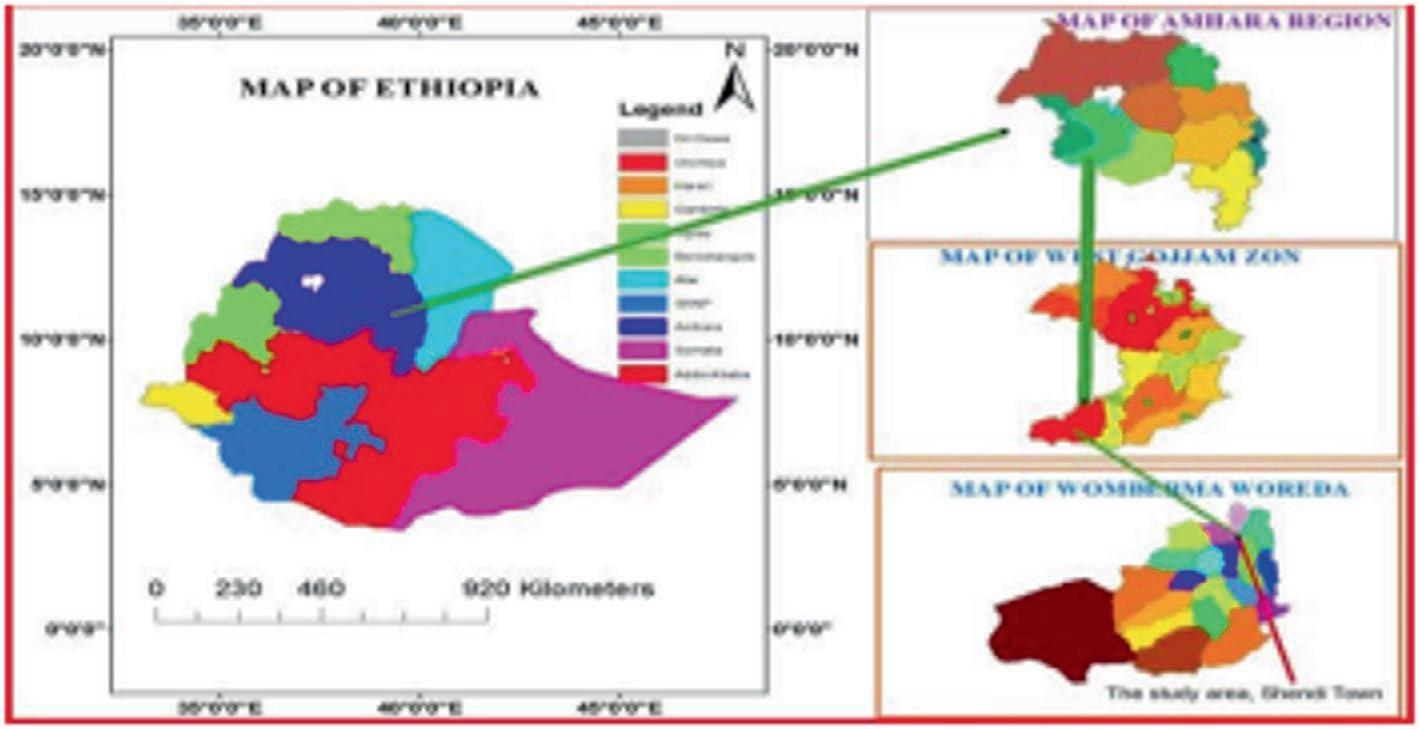
Map of the study area. *Source:* ArcGIS (2022).

The study area employs a mixed farming system, primarily focused on crop production and livestock rearing. Agriculture in this region relies heavily on rainfall, with limited use of irrigation. As reported by [12], out of the total land area, 921,587 ha are suitable for irrigated agriculture, whereas the remainder is cultivated through rain‐fed methods, indicating limited expansion of small‐scale irrigation practices. The primary crops grown in the area include maize, wheat, teff, and barley, which serve as both staple food and cash crops. Additionally, crops such as chickpeas, peas, oil Niger, and beans are cultivated. The area also produces various fruits, including bananas, mangoes, avocados, oranges, and lemons, which are the dominant fruit crops. Rural households derive most of their income from crop sales, followed by the sale of sheep and goats [13]Investigating this particular region provides a perfect study to determine why institutional and socioeconomic challenges prevent technological adoption in a region otherwise provided with outstanding environmental and agricultural potential (Figure 1).

The study utilized cross‐sectional primary data collected during the 2024/2025 cropping season through structured questionnaires, which were administered to respondents to gather relevant information. Given the study′s focus on identifying and describing the factors influencing the adoption of the improved maize BH660 variety among smallholder farmers in Womberma district, the study uses an integrated descriptive and explanatory research strategy in order to methodically untangle these adoption patterns. Descriptive statistics are used to analyze the socioeconomic attributes of the residences, whereas binary logistic regression, an econometric technique, is simultaneously used to determine the causal factors. This dual approach guarantees that the research goes beyond simple description to offer solid, empirically supported explanations of smallholder economic behavior. The target population included both adopters and nonadopters of the improved maize seed within the smallholder farming community. The study respondents were chosen using a multistage sampling design. Womberma district was purposefully chosen from the West Gojjam Zone for the first stage because of its great potential for maize production and the deliberate introduction of the BH660 variety by local extension services. The following steps relied solely on probability sampling to reduce the possibility of purposeful selection bias. In the second phase, a lottery was used to choose five kebeles (the lowest administrative units) at random from the district′s 25 kebeles that produced maize. In the third stage, 370 households were chosen using systematic random selection proportionate to each kebele′s population size from a sampling frame of all 9763 active farming households across the five chosen kebeles by using the formula from [14], with a 5% margin of error and a 95% confidence level. This methodological approach ensured a representative and systematic analysis of the factors affecting the adoption of improved maize varieties among smallholder farmers.

The sampling size for this study was determined by using the formula, as indicated in [14]. This study used the following formula to calculate sample size:

n=z2⋅p⋅1−p⋅Ne2N−1+z2⋅p⋅q=1.962⋅0.5⋅10.5−⋅97630.05297631−+1.962⋅0.5⋅0.5=369.653370~.

Binary logistic regression analysis was employed to estimate the determinants of farmers′ willingness to adopt the improved maize seed variety BH660, utilizing the logit model. The logit model was chosen because of its mathematical simplicity and the direct interpretability of its parameter estimates as odds ratios, even though both the logit and probit models are appropriate for binary outcomes. The logit model is computationally efficient and frequently chosen in econometric studies of agricultural technology adoption since it is based on a typical logistic distribution, as opposed to the probit model, which assumes a normal distribution [15].

In this study, the researcher aims to investigate the factors influencing the adoption of improved maize seed (BH660) among smallholder farmers. To analyze the data involving binary outcomes, the logit model is selected due to its comparative mathematical simplicity. The binary logistic probability model is specified econometrically as follows:

(1) Pi=EY=1Xi=11+e−β0+β1Xi,

where *P*_i_ represents the probability of a farmer being an adopter and *X*_i_ denotes the data reflecting individual preferences (with values of 1 or 0). By substituting *β*_0_ + *β*_1_ *X*_i_ with *Z*_i_, Equation (2) is derived:

(2) Pi=11+e−zi.

Here, *Z*_i_ ranges from −∞ to +∞, whereas *P*_i_ ranges between 0 and 1. The probability of nonadoption is expressed as 1 − *P*_i_ (Equation 3):

(3) 11−11+e−zi==Pi.

The odds ratio, which is the ratio of the probability of adoption to nonadoption, is given by

(4) Pi1−Pi=11/+e−zi1−11/+e−zi=1e−zi=ezi.

Taking the natural logarithm of the odds ratio yields the logit model:

(5) Li=1nPi1−Pi=Zi=β0+β1X1 +βnXn+Ui,

where *P*_i_ is the probability of adopting improved maize seed (BH660), ranging from 0 to 1; *Z*_i_ is a function of explanatory variables *X*; *β*_0_ is the intercept; *β*_1_, *β*_2_, ⋯, *β*_n_ are the slopes of the equation; *L*_i_ is the log of the odds ratio; *X*_i_ represents the vector of farmers′ characteristics; and *U*_i_ is the error term. This model provides a robust framework for understanding the determinants of adoption behavior among smallholder farmers.

In the context of a logit model, odds and odds ratios are crucial concepts. Odds represent the ratio of the probability of an event occurring to the probability of it not occurring, whereas the odds ratio is the ratio of two odds, essentially comparing the likelihood of one outcome to another. Equation (4) illustrates the proportion of probabilities between adopters and nonadopters, representing the odds of proportion. It is essential to recognize that probability, odds, and logit are interconnected concepts, offering different perspectives on the same phenomenon [16].

Furthermore, the marginal effect was computed to quantify the actual influence of each variable on the decision probability. The marginal effect measures the change in predicted probabilities when a binary independent variable shifts from 0 to 1, holding other variables at their means, or reflects the change in the response variable due to a unit change in a continuous independent variable, keeping other variables constant.

The binary logit econometric model is represented as follows:

(6) Zi=β0+∑βiXi+Ui.

Equation (6) was employed to identify key factors influencing the adoption of improved maize seed BH660 among smallholder farmers, where *Z*_i_ denotes the probability of adopting improved maize seed BH660 , *β* represents the parameters of the explanatory variables, and *U*_i_ is the error term. The error term encompasses unobserved factors affecting adoption that fall outside the researcher′s scope. The dependent variable *Z* is binary, with *Z* = 1 indicating adoption and *Z* = 0 indicating nonadoption. This model provides a comprehensive framework for analyzing the determinants of improved maize seed adoption among smallholder farmers.

In addition to socioeconomic factors of enhanced maize variety (BH660) adoption, the model includes rigorous institutional proxies to reduce endogeneity and omitted variable bias. Annual income, loan availability, and farmer attitude are examples of variables that exhibit simultaneous causality; over time, technology adoption can simultaneously increase income and improve attitudes. The study uses exogenous control variables to absorb unobserved household variance in order to isolate these effects. Examples of these indicators include walking distance to primary markets and extension training availability. Model stability against endogeneity‐induced bias is confirmed by postestimation specification diagnostics, such as the Link test and Hosmer–Lemeshow goodness‐of‐fit test Table 1 summarizes the variables, detailing their operational measurements, expected signs (+/−), and the underlying theoretical justifications guiding the study′s hypotheses.

**Table 1:** Summary of explanatory variables.

Variable	Description	Expected signs	Theoretical justification
Educational level	1 if the household head is literate (can read and write); 0 otherwise	+	Education enhanced the ability to understand technical agricultural extensions, instructions, and ideal application rates
Gender	1 if the household head is male; 0 otherwise	+	Male heads typically have better access to and control over productive resources, labor, and formal institutional channels due to customary structural biases
Family size	Total number of individuals living in the household (continuous)	±	Positive: Proxies labor availability
			Negative: Increases dependency ratios and consumption burdens, squeezing cash reserves
Marital status	1 if the household head is currently married; 0 otherwise	±	Whereas single heads may encounter severe labor limits or more risk aversion (−), married heads may enjoy poolable agricultural labor and collaborative decision‐making (+)
Farm experience (year)	Number of years the respondent has been engaged in farming (continuous)	+	Experience increases overall farm management competency and lowers subjective uncertainty about climatic volatility
Access to market information	1 if a respondent has market access; 0 otherwise	+	Reduces asymmetric market risks and gives farmers confidence that any excess production from the upgraded variety may be profitably sold
Access to fertilizer	1 if there is access to fertilizer; 0 otherwise	+	Adoption of high‐yield maize varieties is directly influenced by supply security; these varieties are highly input‐responsive and complementary to fertilizer
Farm land size (hectare)	Continuous (total land area cultivated by the household)	+	Smallholders can assign trial plots for new cultivars without jeopardizing household food security, thanks to larger landholdings
Distance from the farmers′ market (km)	Continuous (shortest distance from the farm gate to the nearest output market)	−	Transaction costs, input transit friction, and structural information asymmetry all increase with distance
Income (annually in ETB)	Total household income from agricultural and nonfarm sources (continuous)	+	Reduces the first barriers to modern input purchases and protects the household from possible crop failure hazards
Access to training	1 if the respondent participated in agricultural extension training; 0 otherwise	+	It upgrades human capital by developing actual knowledge of BH660′s agronomy, planting depth, and precise spacing
Attitude of respondents on farmers′ adoption	1 if the respondent views the new technology as beneficial; 0 otherwise	+	Adoption intentions are immediately triggered by a positive cognitive evaluation of the relative advantage of the technology, which also lowers perceived risk
Number of livestock	Continuous (total livestock ownership measured in tropical livestock units)	+	Represents riches in liquid money that eases seasonal cash flow issues while purchasing expensive commercial supplies
Access to credit	1 if the respondent has access to formal or informal credit services; 0 otherwise	+	Relieves immediate financial constraints during the crucial windows for planting input purchases and preharvest

## 3. Results and Discussion

This section explores the empirical factors influencing smallholder farmers′ adoption of improved BH660 maize varieties in the study area. A logit regression model was used for estimation, with the dependent variable, adoption status, defined as a binary outcome (1 for adoption, 0 otherwise). It is modeled as a function of multiple independent variables, such as socioeconomic, institutional, and farm‐specific factors. Key variables include marital status, gender, income, farm size, experience, access to credit, market information, and training. The model captures the likelihood of adoption while accounting for unobserved factors through the error term. Detailed descriptions of these variables are provided below.

This study compares adopters (*N* = 190) and nonadopters (*N* = 180) using chi‐square (*χ* ^2^) and *t*‐tests to investigate the factors influencing farmers′ adoption of agricultural techniques. The analysis sheds light on the institutional and socioeconomic elements that influence adoption patterns. The main conclusions show that adoption is highly impacted by factors such as gender, educational attainment, training availability, and market knowledge. Compared with females, males are more likely to adopt agricultural advances (*χ* ^2^ = 50.2263^∗∗∗^). Similarly, the adoption rate is higher for literate farmers than illiterate ones (*χ* ^2^ = 62.1161^∗∗∗^). Furthermore, adoption decisions are substantially influenced by market information (*χ* ^2^ = 24.8817^∗∗∗^) and training access (*χ* ^2^ = 47.2101^∗∗∗^), underscoring the importance of knowledge dissemination in enhancing farming methods.

Adopters and nonadopters differ significantly in economic characteristics such as farm experience (−4.6821 ^∗^), annual income (−2.2517 ^∗∗∗^), and farmland size (−2.8329 ^∗∗∗^). Adoption is more likely to occur among farmers with larger farmlands and more years of experience. The lack of statistically significant differences in family size, livestock ownership, and distance from farmers′ cooperatives, however, suggests that these characteristics have less of an effect on adoption behavior. The results highlight how crucial market possibilities, extension service accessibility, and education are in promoting the use of agricultural technology. To increase adoption rates, policymakers and agricultural development initiatives should concentrate on expanding market access and training possibilities. For rural farming communities, strengthening these sectors can result in higher revenues, greater productivity, and better living conditions (Table 2).

**Table 2:** Summary of variables by adoption status.

Variable	Adoption status		Total (*N* = 370)	*χ*2 value/*t*‐value
	Adopters (*N* = 190)	Nonadopters (*N* = 180)		
Gender				
Male	68.62	31.67	68.48	50.23 ^∗∗∗^
Female	31.38	68.33	31.52	
Marital status				
Married	50.06	37.78	44.57	6.57
Single	48.94	62.22	55.43	
Level of education				
Literate	65.96	25	45.92	62.12 ^∗∗∗^
Illiterate	34.04	75	54.08	
Attitudes of respondents toward participation				
Good	70.21	28.33	49.73	64.51
Bad	29.79	71.67	50.27	
Access to fertilizer				
Yes	52.7	49.4	51.1	4.90 ^∗∗^
No	47.3	50.6	48.9	
Access to credit				
Yes	51.6	50.6	51.1	0.04
No	48.4	49.4	48.9	
Access to training				
Yes	69.7	33.9	52.2	47.21 ^∗∗∗^
No	30.3	66.1	47.8	
Access to market information				
Yes	67.0	41.1	54.3	24.88 ^∗∗∗^
No	33.0	58.9	45.7	
Age	40.3 (16.12)	41.2 (15.6)	40.7 (15.8)	0.55
Family size	5.53 (3.25)	4.97 (2.95)	5.26 (3.11)	−1.71
Farm experience	22.46 (14.95)	15.62 (12.94)	19.11 (14.40)	−4.6821 ^∗∗∗^
Annual income	60,173.18	44,986.9	52,745.1	−2.25 ^∗∗∗^
Farmland size	1.009	0.736	0.876	−2.83 ^∗∗∗^
Livestock holding	7.787	8.639	8.203	1.06
Distance from the farmers′ cooperative	9.17	9.38	9.27	0.24

*Note:* Source: field survey, 2025. *** and ** indicate statistical significance at the 1% and 5% levels, respectively.

Important econometric hypotheses were examined prior to logit model estimation in order to guarantee model dependability. The researcher looked for bias from missing variables and mistakes in the model formulation. The outcome, verifying that there were no missing variables or definition mistakes in the model. Goodness‐of‐fit (Hosmer–Lemeshow test) results show that the model fits well. It would appear from this that the explanatory factors adequately account for changes in the dependent variable. Continuous variables were tested using the variance inflation factor (VIF), and dummy variables were examined using contingency coefficient (CC) analysis; both tests revealed no multicollinearity problems. The Breusch–Pagan/Cook–Weisberg test for heteroscedasticity indicates that there is no heteroscedasticity, indicating that error variances are consistent across data.

In this section, this study tries to examine empirical factors that determine the adoption of the improved maize BH660 variety among smallholder farmers within the study area. The logit regression analysis was employed for estimation purposes. The dependent variable “adoption status” is a function of many independent variables. In this study, the major independent variables used to include the dependent variable are described below.

The econometric analysis of factors influencing smallholder farmers′ adoption of the improved maize variety BH660, using a logit model, revealed that the model was statistically significant at the 1% level (prob > *χ* ^2^ = 0.0000), with a strong *χ* ^2^ value confirming its validity and reliability. The model correctly classified 79.08% of observations, accurately predicting 80.85% of adopters and 77.2% of nonadopters. Key variables such as gender, education, training, market knowledge, and farmers′ attitudes were statistically significant at the 1% level, positively influencing adoption, whereas farm experience had a positive impact at the 10% significance level. Conversely, distance from input markets, livestock units, and loan availability negatively but insignificantly affected adoption. Other factors like family size, fertilizer access, annual income, marital status, and farmland size had positive but statistically insignificant effects on adoption. Overall, the study highlights the importance of education, training, and market awareness in promoting the adoption of improved maize varieties among smallholder farmers (Table 3).

**Table 3:** Maximum likelihood estimates of the binary logit model.

Explanatory variable	Marginal effect	Odds ratio	Estimated coefficient	Standard error	*p* value	95% conf. interval
Family size	0.0132	1.0542	0.0528	0.0459	0.251	−0.0372312; 0.1427362
Gender (female = 0)	0.2535	2.8250	1.0385 ^∗∗∗^	0.3409	0.002	0.3704305; 1.70658
Edu_level (0 = illiterate)	0.2819	3.2051	1.1647 ^∗∗∗^	0.3364	0.001	0.5053163; 1.824155
Marital status (0 = single)	0.0626	1.2857	0.2513	0.2888	0.384	−0.3147779; 0.8174576
Farm land size	0.0129	1.0530	0.0517	0.2336	0.825	−0.4060962; 0.5094128
Annual income	5.79e − 09	1.000	2.32e − 08	2.24e − 06	0.992	−0.437e − 06; 4.441e − 06
Access to training (0 = no)	0.2553	2.8433	1.0449 ^∗∗∗^	0.2887	0.000	0.4791665; 1.610751
Access to fertilizer (0 = no)	0.0524	1.2339	0.2102	0.2917	0.471	−0.3615056; 0.781835
Access to credit (0 = no)	−0.0725	0.7474	−0.2912	0.2995	0.331	−0.8782931; 0.2958913
Farm experience	0.0079	1.0322	0.03173 ^∗∗^	0.0152	0.037	0.0019249; 0.0615337
Market information (0 = no)	0.2837731	3.2172	1.1685 ^∗∗∗^	0.2927	0.000	0.5948034; 1.742188
Distance form market	−0.0045	0.98204	−0.0181	0.0174	0.296	−0.0521325; 0.0158831
Attitude (0 = negative)	0.4348332	6.4735	1.8677 ^∗∗∗^	0.2952	0.000	1.289186; 2.446246
Livestock unit	−0.0044	0.9827	−0.0175	0.0178	0.327	−0.0523976; 0.0174604
Cons	—	0.0233	−3.7605	0.5772	0.000	−4.891893; −2.629199
Sensitivity 80.85% No. of observation 370 Pseudo *R* ^2^ 0.3727	Specificity 77.2%		Correctly classified		79.08%	
			LR chi‐square (*p* value)		190.08 (0.0000)	
			Log likelihood		−159.94893	

*Note:* Source: model output (2025).

^∗∗∗^Statically significance at 1%;  ^∗∗^statically significance at 5%.

In terms of marginal effect, the model is as follows:

(7) Adoption status=0.010.061.045.79090.01famsize+marst+gen+e−inc+farmlsize +0.010.430.0050.280.28farmexp+att−dm+edul+accmktinf +0.260.070.050.004acctrn−accredit+acf−tlu.

The statistical significance of the main predictors in the model is explicitly supported by the confidence intervals. A statistically significant positive effect on the outcome variable (*p* < 0.01) is confirmed by the 95% confidence intervals for gender (0.37–1.71), education level (0.51–1.82), access to training (0.48–1.61), market information (0.59–1.74), and attitude (1.29–2.45), which are all positive and do not include zero.

Gender, education, training, farmers′ attitudes, farm experience, and access to market information are critical factors influencing the adoption of the improved maize BH660 variety among smallholder farmers. Econometric analysis reveals that gender significantly affects adoption, with male farmers being 25.3% more likely to adopt the variety compared with their female counterparts at a 1% significance level, highlighting the need to address gender disparities in agricultural technology adoption [17]. Education also plays a pivotal role, as the likelihood of adoption increases by 28.2% when farmers transition from illiteracy to literacy, underscoring the importance of education in enhancing farmers′ ability to access and understand new technologies [6]. Similarly, training significantly boosts adoption, with trained farmers being 25.5% more likely to adopt the improved maize variety, as it enhances their capacity to embrace innovative practices [18]. Farmers′ attitudes toward the technology are equally influential, with those holding positive attitudes being 43.5% more likely to adopt the variety [18]. Farm experience further contributes to adoption, with each additional year of experience increasing the likelihood of adoption by 0.8%. Finally, access to market information is crucial, as it enables farmers to make informed decisions, negotiate better prices, and protect themselves from unfair trading practices, resulting in a 28.4% higher probability of adoption among those with access to such information [19]. These findings emphasize the need for targeted interventions to address these factors and promote the widespread adoption of improved agricultural technologies.

## 4. Conclusions and Recommendations

This study examined the determinants of the adoption of improved maize BH660 varieties among smallholder farmers in the rural West Gojjam Zone, using primary cross‐sectional data collected from 370 respondents. A multistage sampling procedure was employed, with sample farmers selected using the probability proportional technique. The binary logit model was applied to analyze the data and identify key factors influencing adoption. Descriptive statistics revealed that variables such as access to market information, gender, marital status, attitude, farming experience, access to training, farmland size, educational level, and annual income were statistically significant in their association with adoption status. In contrast, access to credit, family size, distance to the input market, access to fertilizer, and TLUs showed no significant association. The binary logit analysis further highlighted that gender, education level, farming experience, attitudes, access to training, and access to market information significantly influenced the adoption of improved maize BH660 varieties. However, marital status, distance from the input market, annual income, access to fertilizer, farmland size, family size, TLUs, and access to credit did not have a statistically significant effect on adoption at any level of significance.

Promoting the adoption of improved maize varieties, such as BH660, among smallholder farmers requires a multifaceted approach that addresses both practical and educational barriers. Introducing advanced technologies that reduce the domestic burden on female smallholder farmers can significantly enhance adoption rates by freeing up time and resources for agricultural activities, thereby empowering women to engage more effectively in farming practices. Simultaneously, improving farmers′ education through targeted adult education programs, training, and awareness campaigns is essential, as literacy enhances their ability to access and utilize information about improved seeds. Collaborative efforts involving NGOs, higher education institutions, and government bodies should organize workshops, public meetings, and mentorship programs to share experiences and highlight the benefits of adopting high‐yielding varieties like BH660. Additionally, improving access to market information through digital tools, radio, and farmer cooperatives can bridge information gaps and foster collective bargaining. Promoting a positive attitude toward new technologies through awareness campaigns and success stories, alongside tailored interventions for less‐experienced farmers, can further accelerate adoption. However, to ensure equitable distribution of benefits, it is crucial to prioritize poor smallholder farmers by providing formal education and accessible training programs, ultimately enhancing productivity and farm efficiency across the board.

**Nomenclature**
CSA: Central Statistical Agency
EARO: Ethiopian Agricultural Research Organization
FAO: Food and Agriculture Organization
GDP: gross domestic product
MoARD: Ministry of Agriculture and Rural Development
TLUs: tropical livestock units

## Author Contributions

The first author, Belete Animaw, is the principal investigator of this study. The second and third authors, Silabat Enyew and Gebyaw Demeke, serve as supervisors and co‐authors. They guided the research process and contributed to editing, as well as to the analysis and interpretation of the data in the manuscript.

## Funding

No funding was received for this manuscript.

## Disclosure

As a result, all sources of materials used in this study have been acknowledged.

## Ethics Statement

We, the authors, declare that this study, “Bridging the Maize Yield Gap: Empirical Evidence on the Adoption of BH660 Hybrid Variety in West Gojjam, Ethiopia,” strictly adhered to international ethical declarations concerning human subjects when performing the field protocols. Before distributing the questionnaire, we got each participating smallholder head′s free and prior verbal informed consent. In order to protect respondent privacy throughout our agricultural surveys in 2024/2025, we also built all data‐gathering technologies to be fully anonymized.

## Conflicts of Interest

The authors declare no conflicts of interest.

## Data Availability

The data that support the findings of this study are available from the corresponding author upon reasonable request.

## References

[1] African Development Bank , Ethiopia Economic Outlook, 2026, African Development Bank Group.

[2] Zegeye M. B. , Meshesha G. B. , and Shah M. I. , Measuring the Poverty Reduction Effects of Adopting Agricultural Technologies in Rural Ethiopia: Findings From an Endogenous Switching Regression Approach, Heliyon. (2022) 8, no. 5, e09495, 10.1016/j.heliyon.2022.e09495, 35647345. 35647345 PMC9130521

[3] Teressa D. , Kibret K. , Dechasa N. , and Wogi L. , Soil Properties and Nutrient Uptake of Maize (Zea mays) as Influenced by Mixed Manure and Blended Inorganic Fertilizer in Haramaya District, Eastern Ethiopia, Heliyon. (2024) 10, no. 16, e35784, 10.1016/j.heliyon.2024.e35784. 39220944 PMC11365320

[4] Abate T. , Shiferaw B. , Menkir A. , Wegary D. , Kebede Y. , Tesfaye K. , Kassie M. , Bogale G. , Tadesse B. , and Keno T. , Factors That Transformed Maize Productivity in Ethiopia, Food Security. (2015) 7, no. 5, 965–981, 10.1007/s12571-015-0488-z.

[5] Ahmed M. H. , Geleta K. M. , Tazeze A. , Mesfin H. M. , and Tilahun E. A. , Cropping Systems Diversification, Improved Seed, Manure and Inorganic Fertilizer Adoption by Maize Producers of Eastern Ethiopia, Journal of Economic Structures. (2017) 6, no. 1, 1–16, 10.1186/s40008-017-0093-8.

[6] Abate T. W. , Analysis of Agricultural Technology Adoption: The Use of Improved Maize Seeds and Its Determinants in Ethiopia, Evidence From Eastern Amhara, Journal of Innovation and Entrepreneurship. (2024) 13, no. 1, 10.1186/s13731-024-00421-4.

[7] Wasiu O. A. and Adebayo S. B. , Determinants of Adoption of Improved Maize Varieties in Osun State, Nigeria, Journal of Agricultural Extension and Rural Development. (2015) 7, no. 3, 65–72, 10.5897/jaerd2014.0605.

[8] Kassa Y. , Determinants of Adoption of Improved Maize Varieties for Male Headed and Female Headed Households in West Harerghe Zone, Ethiopia, International Journal of Economic Behavior and Organization. (2013) 1, no. 4, 33–38, 10.11648/j.ijebo.20130104.11.

[9] Geyo G. B. , Yirga C. , Mohammed J. H. , and Jaleta M. , Maize Varietal Adoption Rate in Ethiopia, the Farmer Self Identification and DNA Finger Printing Approaches, Journal of Natural Sciences Research. (2019) 9, no. 2, 6–25, 10.7176/jnsr/9-2-02.

[10] Geffersa A. G. , Agbola F. W. , and Mahmood A. , Improved Maize Adoption and Impacts on Farm Household Welfare: Evidence From Rural Ethiopia, Australian Journal of Agricultural and Resource Economics. (2022) 66, no. 4, 860–886, 10.1111/1467-8489.12489.

[11] Abate T. M. , Mekie T. M. , and Dessie A. B. , Analysis of Speed of Improved Maize (BH-540) Variety Adoption Among Smallholder Farmers in Northwestern Ethiopia: Count Outcome Model, Heliyon. (2022) 8, no. 10, e10916, 10.1016/j.heliyon.2022.e10916, 36247130. 36247130 PMC9557903

[12] Worku C. , Determinants of Food Security Status of Household in West Gojjam Zone, Ethiopia, Food Science & Nutrition. (2023) 11, no. 10, 5959–5966, 10.1002/fsn3.3527, 37823110. 37823110 PMC10563729

[13] Alamneh T. , Mada M. , and Abebe T. , The Choices of Livelihood Diversification Strategies and Determinant Factors Among the Peri-Urban Households in Amhara Regional State, Ethiopia, Cogent Social Sciences. (2023) 9, no. 2, 10.1080/23311886.2023.2281047.

[14] Kothari C. R. , Research Methodology: Methods and Techniques, 2009, 2nd edition, New Age International Publishers.

[15] Gujarati D. , Basic Econometrics, 2004, 4th edition, The McGraw−Hill Companies.

[16] Menard S. , Applied Logistic Regression Analysis, 2002, 2nd edition, SAGE Publications, 10.4135/9781412983433.

[17] Gebre G. G. , Isoda H. , Rahut D. B. , Amekawa Y. , and Nomura H. , Gender Differences in the Adoption of Agricultural Technology: The Case of Improved Maize Varieties in Southern Ethiopia, Women′s Studies International Forum. (2019) 76, 102264, 10.1016/j.wsif.2019.102264, 31853161. PMC6894305 31853161

[18] Gebre A. , Determinants of Farmer Choice of Milk Market Outlet Channel in Eastern Zone of Tigray, Ethiopia, African Journal of Rural Development and Agricultural Economics. (2024) 6, no. 1, 671–681.

[19] Shiferaw B. , Prasanna B. M. , Hellin J. , and Bänziger M. , Crops That Feed the World 6. Past Successes and Future Challenges to the Role Played by Maize in Global Food Security, Food Security. (2011) 3, no. 3, 307–327, 10.1007/s12571-011-0140-5.

